# Progress of basic research in Parkinson’s disease in China: data mini-review from the National Natural Science Foundation

**DOI:** 10.1186/2047-9158-2-18

**Published:** 2013-08-30

**Authors:** Heqi Cao, Gang Chen, Erdan Dong

**Affiliations:** 1 Department of Medical Science, National Natural Science Foundation of China, Beijing 100085, China; 2 Department of Neurosurgery, The First Affiliated Hospital of Soochow University, Suzhou, Jiangsu Province, China

## Abstract

This review is to analyze the role of National Natural Science Foundation of China (NSFC) on the development of basic research of Parkinson’s disease from 1990 to 2012. Data on the total number of projects and funding of NSFC allocated to Parkinson’s disease, as well as hotspots in western countries, papers published, awards, personnel training, subject construction were collected, and the role of NSFC on other sources of funding was evaluated. Over the past 23 years, a full range of continuous funding from NSFC has led to fruitful results and a strong impetus to the progress of basic research of Parkinson’s disease.

## Review

Parkinson’s disease (PD) is a common neurodegenerative disease observed in the elderly. The latest epidemiological survey shows the total prevalence of PD to be about 160/100,000, but it is above 1.7% among individuals over the age of 65. In China, there currently over 2 million PD patients, and this number continues to increase as the population ages [1,2]. Due to its high prevalence, high rate of disability, and chronic disease course, PD has become a matter of scientific concern as well as a social problem in the fields of demography and health. The application codes for PD from National Natural Science Foundation of China (NSFC) are H0912 (neurodegeneration, neuroregeneration, and related diseases) and H0904 (movement regulation and movement disorders). Here 137 PD-related projects that addressed these issues over the past 23 years (1990–2012) were found in the NSFC database and analyzed. PD projects funded by the E.U. and the U.S. National Institutes of Health (NIH) were also considered for comparison purposes. To perform a comprehensive analysis on funding strategies and hotspots of NSFC in the field of PD, priority PD projects performed over the past 23 years are reviewed regarding their funding and results, and potential problems are also discussed.

### Hotspots in NIH and E.U.-funded studies of PD

PD is the most common neurodegenerative disease in Europe and the United States, and it has received a great deal of attention in Western countries. In the U.S., data from 2003–2005 show that funding for PD-related research added up to more than one billion U.S. dollars, including investment from private industry. Because of this huge financial investment, more than ten types of clinical drugs for the treatment of PD have been released in the past 20 years. These include pramipexole, ropinirole, tolcapone, entacapone, apomorphine, rasagiline, selegiline, carbidopa/levodopa/entacapone, selegiline sublingual, and rotigotine. In 2007, worldwide rankings in PD investment were as follows: North America accounted for 52% (47% from the United States, 5% from Canada), the E.U. accounted for 38% (14% from the U.K. and 24% from other countries), and Japan accounted for 5%. The total amount of PD investment in China is difficult to estimate because data are scattered across the Ministry of Science and Technology, the Ministry of Health, NSFC, and health research institutions at different local levels. However, it is clear that, compared to Europe and the United States, investment in PD-related scientific research in China is still limited [3].

Funding information was retrieved from the NIH website (http://report.nih.gov/). Data showed that, in 2011, NIH funded a total of about 620 projects on PD research, amounting to 151 million U.S. dollars. Funded hotspots included a large number of prospective clinical trials and studies on new drug development, genetic background studies based on a variety of genes, proteins, signaling pathways, and mitochondrial dysfunction, deep brain stimulation (DBS) studies based on new technologies, studies on environmental risk factors and early diagnosis of PD, and studies on cognitive dysfunction in PD patients. During the execution of Research Framework-program 7 (http://cordis.europa.eu/) (2007–2013), E.U. member states and the European Commission invested in a total of about 120 PD research projects, and the research hotspots concentrated in PD related drug development and clinical trials, cell transplantation for treatment of PD, gene therapy of PD, the relationship between lysosomal dysfunction and the pathogenesis of PD, and the relationship between neuroimmunology and PD.

### Brief analysis of NSFC funding of PD-related research projects (1990–2012)

#### Overview

**Figure 1:**
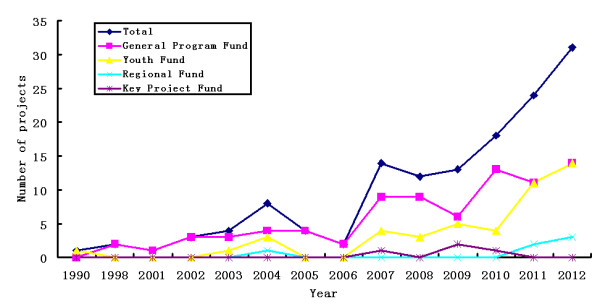
Trend of number of projects during the past 23 years, which showed that NSFC fund more basic studies in PD field year by year.

**Figure 2:**
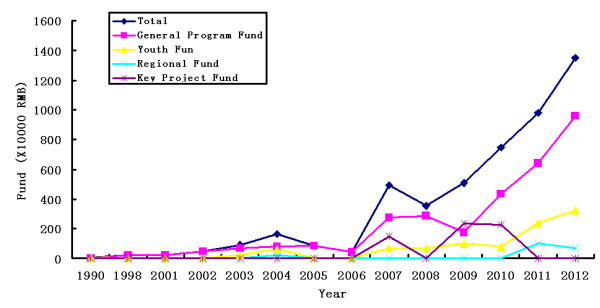
Trend of money funded in PD research during the past 23 years, which showed that NSFC improve the financial intensity in PD field year by year.

From 1990 to 2012, NSFC funded a total of 137 PD-related research projects. Among these, 81 (59%) were funded by the General Program Fund, 46 (34%) by the Youth Fund, 6 (4%) by the Regional Fund (including the special fund), and 4 (3%) by the Key Project Fund (Figure 1). Over the past 23 years, the total funding amounted to 49.04 million RMB, among which the General Program Fund accounted for 31.35 million RMB (64%), the Youth Fund 9.69 million RMB (20%), the Regional Fund (including special fund) 1.86 million RMB (4%), and the Key Project Fund 6.14 million RMB (12%) (Figure 2). Regarding the funding trend, during the 20 years following 1990, the number of funded PD-related projects has been in the single digits every year and the amount of total annual funding has been less than 2 million RMB. However during the past six years, both the number of funded PD projects and the amount of funding have grown substantially. In 2007, the number of funded projects was 14 (a total of 4.95 million RMB), and in 2013, the number was 31 (a total of 13.49 million RMB). Over the past six years, the number of funded PD projects increased by 120% and the total amount of funding increased by 173%. This shows that the NSFC has come to pay more attention to funding PD-related studies recently. Meanwhile, the number of neurologists, neurosurgeons, and neurobiologists applying for grants for PD-related studies has also increased every year.

#### Funding hotspots and fields

**Figure 3:**
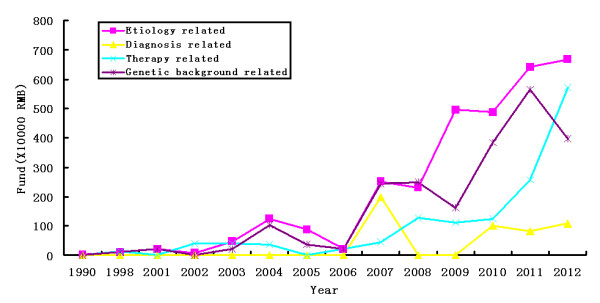
NSFC Funding hotspots and fields in PD research, which demonstrated that the main region for NSFC projects were about etiology and genetic studies in the past 23 years.

Over the past 23 years, the NSFC funded a total of 83 (61%) projects on PD pathogenesis, 11 (8%) on diagnosis, and 42 (51%) on treatment. A total of 30.92 million RMB (62%) was spent in support of studies on etiology, 4.90 million RMB (10%) on diagnostic techniques, and 13.87 million RMB (28%) on treatment. Among funded etiology studies, 63 (46%) of the projects focused on the genetic background of PD and protein function abnormalities, and the total amount of funding was 22.10 million RMB (45%), accounting for the majority of NSFC funding for PD-related studies (Figure 3). These studies investigated PD caused by genetic mutations, PD caused by abnormalities in signaling pathways, the relationship between immune-mediated inflammation and PD, and the relationship between iron and PD pathogenesis. In the field of PD diagnosis, funded projects mainly focused on finding early biomarkers and establishing an early warning system for PD. In the field of treatment, 23 (17%) of the funded projects were related to the development of new drugs covering a total of 7.69 million RMB (16%); 11 (8%) were studies on PD treatment using stem cells, covering a total of 4.46 million RMB (9%); 8 (6%) evaluated PD treatment using electric stimulation, covering a total of 2.55 million RMB (5%).

#### Key projects in PD research

Since 1990, the NSFC funded a total of 4 (3%) Key Projects in the field of PD research (Table 1), and the total amount of funding was 6.14 million RMB (13%). These projects were regionally balanced regarding locations of the laboratories that carried out the studies. One project was conducted in eastern China at Shanghai Jiaotong University (led by Weidong Le and performed on biological markers for early diagnosis of Parkinson’s disease and gene-targeted therapy), one was conducted in southwestern China at the Fourth Military Medical University (led by Guodong Gao and performed on mechanisms underlying synchronous oscillation and stochastic resonance of abnormal symptoms of Parkinson’s disease), one was conducted in northern China at Qingdao University (led by Junxia Xie, and performed on mechanisms and prevention of selective aggregation of iron in substantia nigra and its specific damage to dopaminergic neurons in Parkinson’s), and one was conducted in southern China at Sun Yat-sen University (led by Mingtao Li and performed on mechanisms underlying GSK-3alpha/beta regulation and its use as a target for the treatment of Parkinson’s disease). This and the projects funded by the Youth Fund and General Program Fund show that NSFC funding in PD has focused on projects that investigated etiology, early diagnosis, and new methods of treating PD.

**Table 1:** Key projects in PD research

**Year**	**PI**	**Affiliation**	**Title**	**Fund (X10000RMB)**
2007	Weidong Le	Shanghai Jiaotong University	Biological markers for early diagnosis of Parkinson’s disease and gene-targeted therapy	150
2009	Junxia Xie	Qingdao University	Mechanisms and prevention of selective aggregation of iron in substantia nigra and its specific damage to dopaminergic neurons in Parkinson’s disease	164
2009	Guodong Gao	The Fourth Military Medical University	Mechanisms underlying synchronous oscillation and stochastic resonance of abnormal symptoms of Parkinson’s disease	170
2010	Mingtao Li	Sun Yat-sen University	Mechanisms underlying GSK-3alpha/beta regulation and its use as a target for the treatment of Parkinson's disease	230

#### Outcomes of funded projects

**Figure 4:**
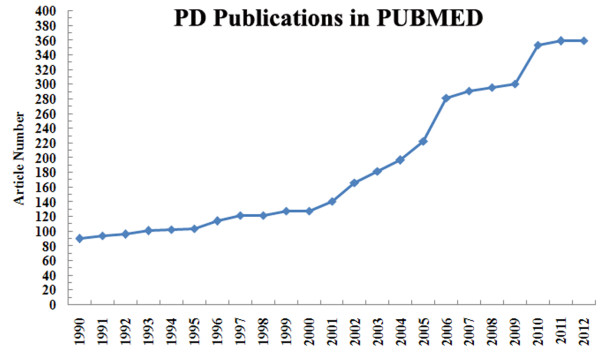
Number of publications titled by ‘Parkinson’s disease’ in PUBMED from 1990 to 2012, which demonstrated that more articles in this field published in the past 10 years.

We searched in PUBMED (http://www.ncbi.nlm.nih.gov/pubmed/) for PD research publications from 1990 to 2012 (Figure 4), which showed that more and more articles have been published in PD field in the past 10 years internationally. At the same time, a search on Web of Science (http://apps.webofknowledge.com/) showed that, through the present, there have been a total of 773 papers with an SCI on PD related-research in China. Among these, 141 (18%) were funded by the NSFC. Especially early on, many Chinese scholars did not note their sources of funding in their papers. In others, the source of funding may have been provided in non-standard formats. This suggests that the actual number of NSFC-funded PD projects may have exceeded 141. The majority of these 141 papers were published in mainstream neuroscience journals. The Xie group in Qingdao University published a paper in *Neurology* in January 2013 stating that, in the temporal lobe tissues of PD patients, the expression levels of proteins related to iron and iron metabolism (DMT1, IRE, TfR1, FPN1, and IRP1) were significantly lower than in healthy controls, but the expression levels of these proteins in the brain tissue of patients with Alzheimer’s disease remained unchanged. This indicates that abnormalities in iron metabolism and iron distribution may be related to PD [4]. The Chen group based in Ruijin Hospital, Shanghai Jiaotong University, published a review in *Progress in Neurobiology* in August 2012 on the significance of kinases and related signaling pathways in the treatment of PD [5]. This group received a total of 14 NSFC grants over the past 23 years and another grant from the National 973 Program (2011CB504104, a basic study on pathogenesis of and on intervention strategies for Parkinson’s disease). This group was granted the first prize Natural Science award conferred by the Chinese Ministry of Education in 2010, and their results had a profound impact on the field of PD research internationally.

### Suggestions and prospects

By reviewing patterns of funding in the field of PD research in China over the past, there were found to be relatively few studies on the surgical treatment of PD. Although, over the past several decades, stereotactic techniques have undergone rapid development, and DBS technology is currently being perfected, there are still a large number of clinical issues that need to be resolved with basic research. More neurosurgeons may apply for studies on the surgical treatment of PD. In addition, current DBS technology was found to have been applied to the treatment of a variety of neuropsychiatric disorders, such as anorexia nervosa [6] and depression [7]. Although NSFC mainly funds basic research, it may also provide financial support for small, highly innovative clinical trials. For example, the explorations performed by Canadian scholars in anorexia nervosa [6] collected data from treatment and follow-ups of only six patients, but the results were considered intriguing enough to be published in *Lancet*. China’s population is gradually aging, and the occurrence of various neurodegenerative diseases such as PD, is significantly correlated with age. For this reason, explorations on the cause, early diagnosis, and treatment of PD must be supported. Strategic guideline that to support basic research, facilitate free exploration, and play a guiding role may allow the NSFC to further strengthen its financial support for PD research, and the field of PD research in China will continue to grow.

## Competing interests

The authors declare that they have no competing interests.

## Authors’ contributions

HC and GC carried out the whole data analysis and drafted the manuscript. ED revised the manuscript. All authors read and approved the final manuscript.
